# Predicting Glioblastoma Recurrence from Preoperative MR Scans Using Fractional-Anisotropy Maps with Free-Water Suppression

**DOI:** 10.3390/cancers12030728

**Published:** 2020-03-19

**Authors:** Marie-Christin Metz, Miguel Molina-Romero, Jana Lipkova, Jens Gempt, Friederike Liesche-Starnecker, Paul Eichinger, Lioba Grundl, Bjoern Menze, Stephanie E. Combs, Claus Zimmer, Benedikt Wiestler

**Affiliations:** 1 Department of Diagnostic and Interventional Neuroradiology, Klinikum rechts der Isar, Technical University of Munich, 81675 Munich, Germany; MarieM.93@gmx.net (M.-C.M.);; 2 Image-Based Biomedical Modeling, Chair for Computer Aided Medical Procedures & Augmented Reality, Technical University of Munich, 85748 Garching, Germany; miguel.molina@tum.de (M.M.-R.); jana.lipkova@tum.de (J.L.);; 3 Department of Neurosurgery, Klinikum rechts der Isar, Technical University of Munich, 81675 Munich, Germany; 4 Department of Neuropathology, Institute of Pathology, Technical University of Munich, 81675 Munich, Germany; 5 Department of Radiation Oncology, Klinikum rechts der Isar, Technical University of Munich, 81675 Munich, Germany; 6 Institute of Innovative Radiotherapy, Helmholtz Zentrum Munich, 85764 Munich, Germany; 7 German Cancer Consortium (DKTK), Partner Site Munich, and German Cancer Research Center (DKFZ), 69120 Heidelberg, Germany

**Keywords:** glioblastoma, DTI, FA, deep learning, recurrence prediction

## Abstract

Diffusion tensor imaging (DTI), and fractional-anisotropy (FA) maps in particular, have shown promise in predicting areas of tumor recurrence in glioblastoma. However, analysis of peritumoral edema, where most recurrences occur, is impeded by free-water contamination. In this study, we evaluated the benefits of a novel, deep-learning-based approach for the free-water correction (FWC) of DTI data for prediction of later recurrence. We investigated 35 glioblastoma cases from our prospective glioma cohort. A preoperative MR image and the first MR scan showing tumor recurrence were semiautomatically segmented into areas of contrast-enhancing tumor, edema, or recurrence of the tumor. The 10th, 50th and 90th percentiles and mean of FA and mean-diffusivity (MD) values (both for the original and FWC–DTI data) were collected for areas with and without recurrence in the peritumoral edema. We found significant differences in the FWC–FA maps between areas of recurrence-free edema and areas with later tumor recurrence, where differences in noncorrected FA maps were less pronounced. Consequently, a generalized mixed-effect model had a significantly higher area under the curve when using FWC–FA maps (AUC = 0.9) compared to noncorrected maps (AUC = 0.77, *p* < 0.001). This may reflect tumor infiltration that is not visible in conventional imaging, and may therefore reveal important information for personalized treatment decisions.

## 1. Introduction

Glioblastoma is the most aggressive primary brain tumor, with a median survival rate of only 15 months despite intensive treatment that usually consists of surgery and subsequent radiochemotherapy [1]. One of the most fatal characteristics of glioblastomas is their ability to diffusely infiltrate the brain tissue, which leads to the commonly accepted assumption that contrast-enhancing tumor margins do not represent true tumor borders [2]. Consequently, most tumor recurrences occur in the peritumoral edema, whereas only about 10% show distant tumor growth at first recurrence [3]. Therefore, much effort has been undertaken to identify means to assess nonenhancing peritumoral edema for tumor infiltration that is not visible in conventional imaging [4,5]. Diffusion-tensor imaging (DTI) has emerged as a promising method to visualize tissue microstructures by modeling displacements of water molecules in different directions using a diffusion tensor. A regularly studied DTI parameter is fractional anisotropy (FA), which reflects the directionality of brain-fiber tracts, and correlates with cell density and proliferation activity [6]. In general, tumor infiltration or invasion is characterized by a variable degree of anisotropy reduction. However, peritumoral edema also results in reduced anisotropy in white matter, which significantly hinders differentiation between pure edema and an actual tumor invasion [7].

In a retrospective study from 2017, Bette et al. detected that, in nonenhancing peritumoral edema, FA values were significantly lower in regions with later tumor recurrence than in regions without. This was measured in comparison to the contralateral, nonaffected side to consider regional differences [8]. However, results of this study and similar ones are controversial due to reproducibility issues [9]. A main reason for this is the susceptibility of DTI to free-water contamination, which impedes analysis of peritumoral edema.

A strategy to reduce this uncontrolled variability is to eliminate the free-water signal stemming from the edema [10], with free water defined as water molecules that do not experience flow and are not restricted by their surroundings [11]. In 2009, Pasternak et al. introduced an algorithm that extracts free water from diffusion MRI, enabling the better estimation of tissue-specific indices, such as FA, in areas of a partial volume effect with cerebrospinal-fluid (CSF) or edema contamination, and more comprehensive fiber tracking in healthy and pathological conditions [12]. This was achieved by fitting a bitensor model for which a mathematical framework was introduced to stabilize the fitting. Since fitting the diffusion tensor in a two-compartment model is an ill-posed problem [13,14], we developed a new method for free-water elimination on the basis of an artificial neural network (ANN) that is independent of the numbers of diffusion shells (*b*-values), and can be retrospectively applied to any diffusion MRI data [15].

In this study, we evaluated the benefit of this novel approach for the free-water correction of DTI data for the prediction of later tumor recurrence from the first preoperative MR scan. Thus, we aimed to discover alterations in the peritumoral edema that are not visible in conventional imaging, but probably indicate tumor infiltration.

Here, we comparatively investigated the prognostic potential of FA with and without free-water correction, as well as changes in mean diffusivity (MD) and tissue-volume fraction estimated by the ANN model. We identified a significant difference in free-water-corrected FA values in regions with later tumor recurrence and those of pure edema. Such differentiation, obtained directly from preoperative MR scans, has the potential to provide crucial information for personalized treatment decisions. Furthermore, analysis did not show any significant prognostic potential of MD changes and estimated tissue-volume fractions.

## 2. Results

### 2.1. Tissue-Volume-Fraction Estimates

**Figure 1:**
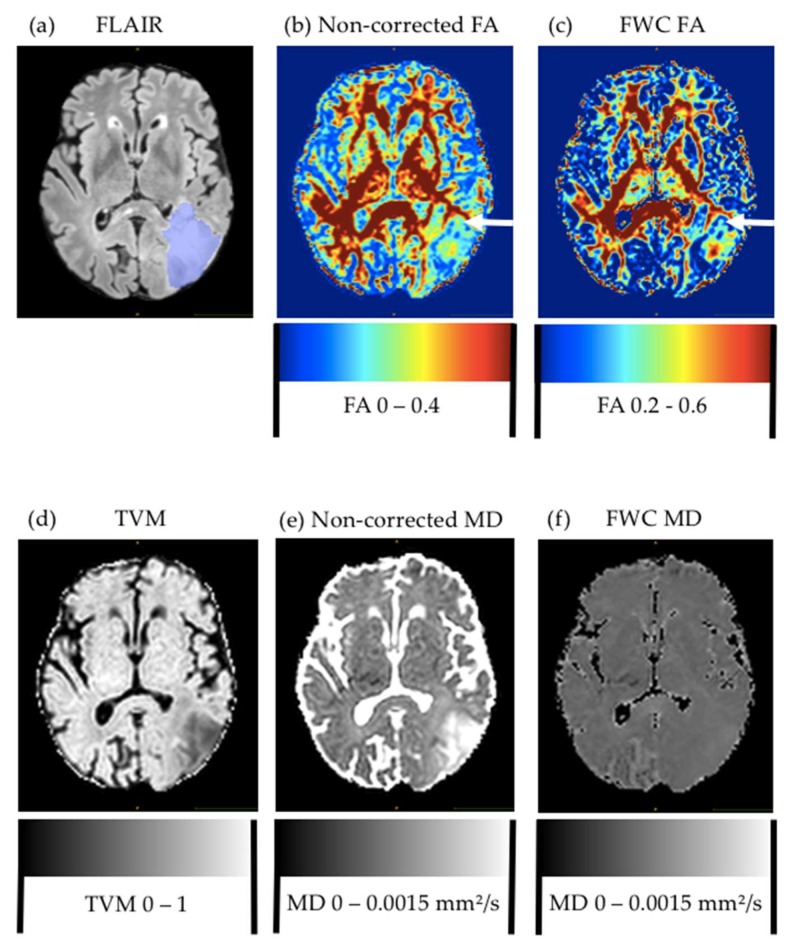
Comparison of metrics for glioblastoma patient. Preoperative FLAIR (**a**) with edema segmentation (overlaid in blue), and (**b**) noncorrected and (**c**) free-water-corrected fractional-anisotropy (FA) maps. Arrow in both FA maps points to area of later tumor recurrence. For improved visualization, display windows were adapted. (**d**) Tissue-volume map with generally lower values in peritumoral edema, but no significant differences between pure edema and edema with later recurrence. Mean-diffusivity map (**e**) before and (**f**) after free-water correction, which noticeably decreased contrast.

**Figure 2:**
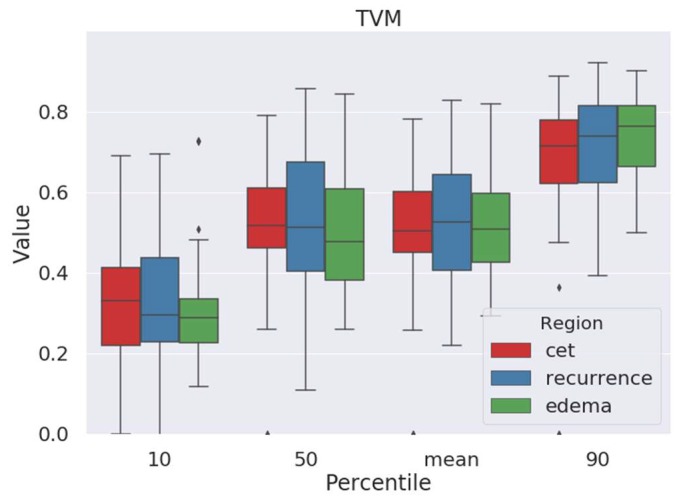
Tissue-volume fractions of contrast-enhancing tumor (red), pure edema (green), and area of peritumoral edema with later recurrence (blue). No significant difference between edema with later tumor recurrence and recurrence-free edema. Diamonds denote outliers.

The ANN we developed was trained to estimate fractions of water and tissue, respectively, in each voxel. Using this information, we generated tissue-volume maps that visualized the tissue fraction in each voxel. We analyzed these data to identify if there were significant differences in the amount of tissue in the area of peritumoral edema on preoperative MR images where later tumor recurrence occurred. However, there were no significant differences in the 10th, 50th or 90th percentile of tissue-volume-fraction values (Table 1, Figure 1 and Figure 2). Thus, we subsequently evaluated parameter maps obtained from both standard and free-water-corrected DTI measurements.

### 2.2. Free-Water-Corrected Mean Diffusivity

**Figure 3:**
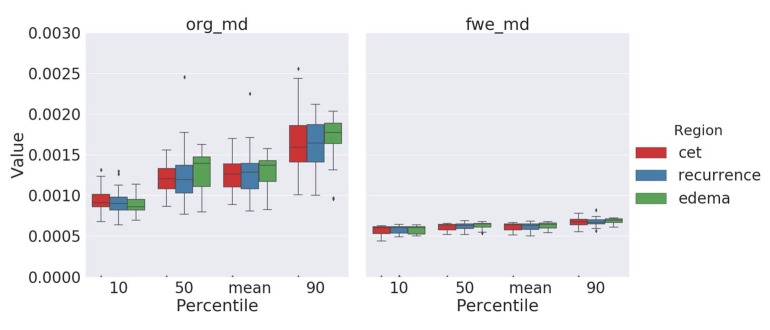
Original mean-diffusivity values (left) and mean diffusivity following free-water elimination (right) of contrast-enhancing tumor (red) and peritumoral edema with (blue) and without (green) later tumor recurrence. Significant differences between pure edema and edema with later recurrence were only observable in the 90th percentile of free-water-corrected MD values. Diamonds denote outliers.

Next, we examined mean-diffusivity (MD) values after performing free-water elimination as described in Section 4. We suspected that, after elimination of the free-water signal, there could be noticeable differences in MD values between areas of contrast-enhancing tumor, pure edema, and edema with later recurrence, respectively. These results are summarized in Table 1 and Figure 3. However, these differences appeared to be rather small and were only significant in the 90th percentile with *p*_90_ = 0.04648400 when comparing pure edema with edema showing later recurrence, with lower MD values in areas of later recurrence. As we expected, there were no significant differences without applying free-water correction (*p*_90_ = 0.16753559).

### 2.3. Fractional-Anisotropy Recovery

Visual comparison of standard and free-water-corrected FA maps revealed a relevant recovery of FA information, especially in areas with large partial volume contamination, such as peritumoral edema and the borders of the ventricles, leading to a visual improvement of FA maps (Figure 1). This recovered new information about tissue-microstructure integrity, previously hidden by the edema, which motivated us to further evaluate the utility of this information for predicting tumor recurrence.

**Figure 4:**
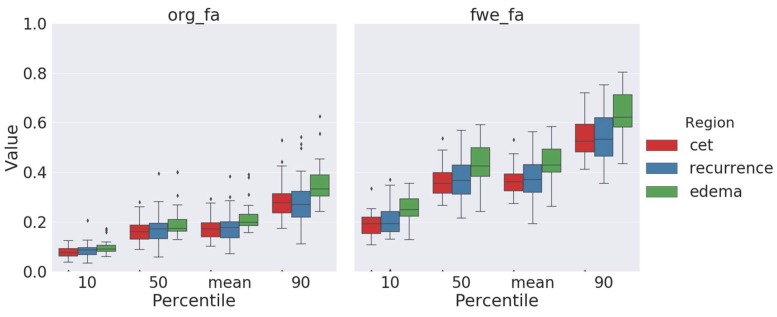
Noncorrected values of fractional anisotropy (left) and FA values after free-water correction (right) comparing contrast-enhancing tumor (red), pure edema (green) and edema showing later tumor recurrence (blue), each for 10th, 50th, and 90th percentile and mean. There are significant differences between recurrence-free edema and edema with later recurrence in all comparisons with free-water corrected FA values. In contrast, the noncorrected FA values only showed significant differences in the 90^th^ percentile. Diamonds denote outliers.

By assessing the peritumoral edema, we found significant differences in free-water-corrected FA maps between areas of recurrence-free edema and areas with later tumor recurrence in all of the three percentiles, with *p*_10_ = 0.00112, *p*_50_ = 0.00314, and *p*_90_ = 0.00007, as well as in the mean values (Table 1 and Figure 4). More precisely, FA values of regions with later tumor recurrence were significantly lower than those of “pure” recurrence-free edema.

In contrast, the original, noncorrected FA maps only showed differences in the 90th percentile, while the 10th and 50th percentiles were not significantly different between both areas (*p*_90_ = 0.0003 vs. *p*_10_ = 0.07515 and *p*_50_ = 0.07908).

**Figure 5:**
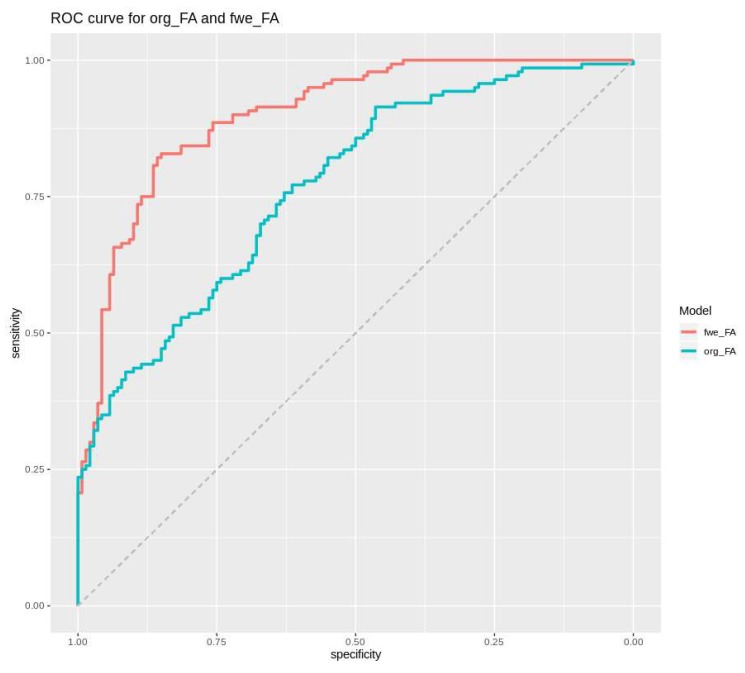
Receiver-operating-characteristic curves for a generalized mixed-effect model to predict later recurrence on the basis of free-water-corrected (FWC) FA values (red curve; AUC = 0.9) or noncorrected FA values (turquoise curve; AUC = 0.77). Both models were threefold cross-validated.

To compare our results with previously published data on the use of FA maps for recurrence prediction [8], we randomly sampled 3 × 3 × 3 mm patches with and without later recurrence and fitted three generalized mixed-effects models from either noncorrected or free-water-corrected (FWC) FA values (or both) to predict areas of later recurrence. The cross-validated area under curve (AUC) for FWC–FA values was 0.9, significantly higher than the AUC for a model based on noncorrected FA values (AUC = 0.77, *p* < 0.0001, DeLong’s test; Figure 5). Consequently, in a model including both FWC and noncorrected FA values, only FWC values were significantly associated with later recurrence.

## 3. Discussion

Free-water-corrected FA maps emerged as a promising tool to better assess peritumoral edema for tumor infiltration. After free-water correction, FA values of regions with later tumor recurrence significantly differed from those of pure edema; areas of later recurrence showed significantly lower FA values, comparable to FA values seen in contrast-enhancing tumors. This may reflect tumor infiltration that is not visible in conventional imaging, and may therefore reveal important information for personalized treatment decisions. In contrast, both free-water-corrected MD values and tissue-volume estimates were not significantly different between areas with and without later tumor recurrence.

In our experiments, we used a two-compartment model to disentangle the “true” diffusion signal from free-water contamination. We hypothesize that improvements we saw in distinguishing areas of later tumor recurrence from recurrence-free areas stemmed from two important effects of free-water correction. First, it shifted variability from the diffusion metrics to the volume-fraction estimates, and second, it eliminated variability induced by the presence of free water by removing the diffusion-isotropic noise from the diffusion signal with the result of revealing actual tissue anisotropy.

Assessing peritumoral edema for tumor infiltration is highly important for improving patient outcomes by enhancing accurate therapy planning. On the one hand, precise surgical planning is crucial for prolonging the progression-free survival of a patient, since gross-total tumor resection was proven to be an important prognostic factor [16]. On the other hand, radiotherapy preserves function and increases survival, aiming to improve local control at a reasonable benefit ratio [17].

In clinical routine, the clinical target volume (CTV) for radiotherapy is defined by a mostly uniform margin of 1.0–2.5 cm set around the area of residual T1 enhancement (if present) and the surgical bed to account for invisible tumor infiltration, following the guidelines of the European Society for Radiotherapy and Oncology (ESTRO) Advisory Committee on Radiation Oncology Practice (ESTRO-ACROP) [18]. This volume is typically slightly modified to reduce the dose to critical structures such as optic nerves, brainstem, pituitary gland, and hippocampus, and another margin of 0.3–0.5 cm is added to account for errors in setup and movement during treatment, resulting in the final planning target volume [17]. Despite intensive treatment, most glioblastomas undergo recurrence. Within 6 months of initializing treatment, 75% show recurrence [19]. This leads to the assumption that there is incomplete tumor coverage by the CTV. We previously showed that deep-learning-derived corrected DTI maps may be used for personalized radiotherapy planning [20].

As demonstrated by Bette et al., we find lower FA values in areas of later tumor recurrence [8]. Through correction of the influence of free water on FA maps, we could better differentiate between areas with tumor infiltration (and later recurrence) and “pure” edema. The utility of this method to better characterize gray matter (and possible tumor infiltration there) and to distinguish white-matter and gray-matter areas in peritumoral edema warrants future studies. In addition, how improved delineation of fiber tracts might benefit neuronavigation and guided resections is a potential future research direction. To facilitate distribution of our method and its application in other centers, we made the source code for our model publicly available (https://github.com/mmromero/dry).

As opposed to previous studies, we unbiasedly analyzed the entire peritumoral edema using tumor segmentation and nonlinear image registration, whereas earlier works focused on individually drawn regions of interest (ROI) in the contralateral hemisphere. Coupled with algorithmic strategies for automated brain-tumor segmentation that we actively develop [21], this opens up exciting possibilities for fully automated analysis of brain-tumor MR images. In order to compare our results to previous reports, we analyzed the potential of FA values to predict later recurrence in mixed-effect models. Compared to Bette et al. [8], who described an area under the curve for manually corrected FA values of 0.893, we report very similar performance for FWC FA values (area under curve = 0.9). However, our method does not require manual correction, therefore allowing unbiased analysis of the entire tumor (thus promising higher robustness compared to manual ROI placement), and can be integrated into fully automated analysis workflows.

In contrast to FA values, MD values did not reveal additional information about the peritumoral edema and later recurrence after performing free-water correction. In general, MD values can perfectly describe isotropic diffusion but not the whole properties of anisotropic diffusion [22]. In most brain tumors, when compared to normal brain tissue, the diffusion coefficient is elevated [7]. Concerning the recurrence prediction and classification of primary brain tumors, prior studies found that there is a decrease in MD or apparent diffusion coefficient (ADC) in areas of peritumoral edema that showed later recurrence [23], as well as decreasing ADC values with an increasing World Health Organization (WHO) grade [24]. This made us investigate potential changes in MD values after free-water elimination. However, we only observed a small difference in the 90th percentile. As expected, MD values were lower in areas of later recurrence. Given that free water is basically the source of contrast in MD, we expected to lose signal through free-water correction. Given this decreased contrast in MD images (see Figure 1), different methods of DTI processing might be more suitable for MD data analysis. More research should be undertaken to detect possible further potential of MD in evaluating tumor infiltration.

Our study has two important limitations. First, it was conducted in a single center. Though the ANN should be able to generalize to DTI data from different centers with comparable performance, this needs to be demonstrated in future multicenter studies. Second, this is retrospective analysis in spite of a prospective observational cohort. Although we included all patients with available data, we cannot exclude a selection bias towards patients with local recurrences.

## 4. Materials and Methods

We investigated 35 glioblastoma cases from our prospective glioma cohort that was approved by the local ethics committee. All patients were scanned in a 3 T whole-body MRI scanner (Achieva, Philips Medical Systems, Best, The Netherlands). The protocol consisted of DTI (2 mm isotropic resolution, TE = 78 ms, TR = 5000 ms) with 32 directions (*b* = 800s/mm^2^) and one nondiffusion-weighted volume (N_b_ = 32), T2 turbo spin echo (T2w), T2-FLAIR, nonenhanced and contrast-enhanced T1 (CE-T1w). All patients underwent gross-total tumor resection following this initial MRI, and all cases were confirmed by histopathological study as IDH-wildtype glioblastoma according to the 2016 WHO classification of brain tumors [25].

The preoperative MR image and first MR scan showing tumor recurrence were semiautomatically segmented into contrast-enhancing and FLAIR-hyperintense areas using an in-house-developed segmentation algorithm (see Figure 1c). Afterwards, segmentation masks were manually corrected where necessary using ITK-SNAP [26]. Next, we nonlinearly coregistered (SyN) the scan showing tumor recurrence to the preoperative MRI using the open-source ANT framework, and warped the segmentation of tumor recurrence (using nearest-neighbor interpolation) onto the preoperative MR [27]. Combining both segmentation masks in the preoperative image space allowed us to objectively extract DTI parameters without the need for subjective and unreliable manual region-of-interest placement.

Free-water correction of preoperative DTI data was performed using a model based on an artificial neural network (ANN), as previously described [15]. In brief, this ANN learns from synthetically generated data (with known truth), a nonparametric forward model that maps free-water partial volume contamination to volume fractions, i.e., it decomposes the measured diffusion signal into a “true” diffusion signal and free-water contamination. The ANN itself consists of an input layer of as many units as the number of acquired *b-*values (N_b_), two hidden layers with N_b_/2 and N_b_/4, respectively, and a single output unit yielding the estimate of tissue-volume fraction. The model is freely available at https://github.com/mmromero/dry.

In DTI, the diffusion tensor basically describes the 3D diffusion phenomenon of water molecules by using a matrix of numbers derived from measurements of at least six or more applied diffusion gradients. After analytical diagonalization of the diffusion tensor, a 3D shape is formed by calculating three eigenvectors that are perpendicular to each other. MD is simply the mean of these three eigenvalues, whereas FA represents a ratio of diffusion coefficients with lower values, describing nearly isotropic diffusion, and higher ones, characterizing extremely anisotropic diffusion restricted along two of the three eigenvector directions [7,22].

Here, robust estimation of tensors by outlier rejection (RESTORE) was employed for tensor estimation, and subsequent MD and FA calculation both from the uncorrected and free-water-corrected DTI data [28].

To apply these methods for tumor-recurrence prediction, we assessed peritumoral edema for alterations in MD and FA values before and after free-water correction. In particular, we automatically extracted the 10th, 50th, and 90th percentile, as well as the mean of MD and FA values (both for original and free-water-corrected DTI data) from areas with and without later tumor recurrence in peritumoral edema. We employed Wilcoxon’s rank-sum test to compare MD and FA values from peritumoral edema with and without later tumor recurrence. In line with analysis by Bette et al. [8], we randomly sampled eight 3 × 3 × 3 mm patches from the perifocal edema of each patient, of which four showed later recurrence and four did not. We fitted three generalized mixed-effect models (with “patient” being the random effect) to predict later recurrence: (a) using only FWC–FA values from the patches, (b) using only noncorrected FA values, and (c) using both values. Threefold cross-correlation was used to minimize bias while fitting the models. DeLong’s test was used to compare correlated receiver-operating-characteristic (ROC) curves. All analyses were done in Python (3.6) and R (3.6), and all scripts used for this analysis are available from the corresponding author upon request.

## 5. Conclusions

Correcting the free-water contamination of DTI using an artificial neural network has the potential to relevantly improve tumor delineation in the first preoperative MR scan, which may not only inform more accurate surgery planning, but also help to generate personalized radiotherapy plans that spare more healthy tissue while allowing for dose escalation in areas that are prone to tumor recurrence. This could possibly lead to prolonged progression-free and overall survival in glioblastoma patients, and highlights the potential of AI-driven image analysis.

## Figures and Tables

**Table 1:** Comparison of different percentiles and mean of tissue volume, mean diffusivity, and fractional anisotropy between pure peritumoral edema and edema with later recurrence without (original values, left) and after free water correction (free-water-corrected, right).

Map	Percentile	*p*-Value Edema vs. Recurrence, Noncorrected FA Maps	*p*-Value Edema vs. Recurrence, Free-Water-Corrected (FWC) FA Maps
**Tissue volume**			
	10th	*n*/a	0.41430
	50th	*n*/a	0.42105
	Mean	*n*/a	0.61763
	90th	*n*/a	0.39444
**Mean Diffusivity**			
	10th	0.30961	0.80062
	50th	0.19837	0.15018
	Mean	0.24728	0.23317
	90th	0.16754	0.04648
**Fractional Anisotropy**			
	10th	0.07515	0.00112
	50th	0.07908	0.00314
	Mean	0.06146	0.0029
	90th	0.00030	0.00007
